# Clinicopathologic and Endosonographic Characteristics of Colon Subepithelial Tumors Discovered Incidentally

**DOI:** 10.3390/diagnostics14050551

**Published:** 2024-03-05

**Authors:** Aryoung Kim, Sung Noh Hong, Dong Kyung Chang, Young-Ho Kim, Ji Eun Kim, Eun Ran Kim

**Affiliations:** Department of Medicine, Samsung Medical Center, Sungkyunkwan University School of Medicine, 81 Irwon-ro, Gangnam-gu, Seoul 06351, Republic of Korea

**Keywords:** colon subepithelial tumor, endoscopic ultrasound, histological finding

## Abstract

Background/Aims: Colonoscopy is commonly used for colorectal cancer screening; therefore, the detection of colon subepithelial tumors (SETs) has also increased. Several research studies have been undertaken to diagnose and treat stomach and rectal SETs. The purpose of this study was to determine a diagnostic point for colon SETs by comparing histological findings with the endoscopic characteristics of colon SETs discovered by chance. Methods: A total 194 patients underwent an endoscopic ultrasound (EUS) for suspicious colon SETs during a colonoscopy from May 2014 to December 2021. A total of 105 colon SETs, which were histologically diagnosed, were finally included. Fisher’s exact test was used to determine the factors associated with malignant SETs. Results: Colon SETs were predominantly present in the right colon (*n* = 73, 69.5%), particularly in the transverse colon (*n* = 32, 30.5%). The majority were smaller than 10 mm (*n* = 88, 83.8%), and they had hard consistencies (*n* = 84, 80%) and exhibited no surface changes (*n* = 96, 91.4%). Most of them were found in the submucosal layers (*n* = 54, 51.4%) and had a hypoechoic pattern (*n* = 56, 53.3%) in the EUS. Of the histologically confirmed cases, only three (3/105, 2.9%) were malignant. Most benign lesions were lipomas, suspected parasitic infections, or lesions caused by various inflammatory reactions, including fibrous/fibrocalcific lesions and necrotic nodules. All soft lesions were benign. Two of the three malignant lesions were adenocarcinomas, and the other was lymphoma. For the malignant SETs, there was a statistically significant alteration in the surface of the tumors (*p* < 0.001), and they were located where the muscularis mucosa layer was included (*p* = 0.008). The potential malignant SETs, granular cell tumors, and neuroendocrine tumors (NETs) had similar features, such as yellowish hypoechoic masses. Colon NETs were only found in the rectosigmoid junction. Parasitic infections and lesions, resulting in various inflammatory reactions, were observed as pale and hard SETs and mostly revealed as mixed echogenic masses located in the muscularis mucosa, submucosa, or multi-layers in the EUS. Conclusion: This study showed that small colon SETs were mostly benign lesions. Despite its rarity, pathological confirmation is crucial in cases where the SET has surface changes and has been located in a position where the muscularis mucosa layer was included on the EUS, due to the risk of malignancy.

## 1. Introduction

Subepithelial tumors (SETs) are raised lesions that occur between the deep mucosa and the serous layer, surrounded by normal mucosa [1]. The reported incidence of colon SETs is increasing due to improvements in colonoscopies and computed tomography (CT) [2,3,4]. Most cases are incidentally discovered during endoscopic screening without specific symptoms, and there is hesitation around performing a forceps biopsy due to potential diagnostic challenges and shape changes caused by the biopsy [5].

Colon SETs include lipomas, lymphangiomas, leiomyomas, and carcinoid and neoplastic lesions, each requiring different treatment and prognoses [6,7]. An accurate differential diagnosis is crucial, but there have been few studies on the diagnostic approaches for colon SETs [2,8]. An endoscopic ultrasound (EUS) provides detailed information on the gastrointestinal wall structure and adjacent organs, making it useful for observing submucosal lesions and their origins [8].

Studies on SETs in the upper gastrointestinal tract and rectum have been published [9,10,11,12,13,14]. However, studies on SETs of the colon are limited, and the study was small, with fewer than 50 patients [2,7,15]. To our best knowledge, there has only been one systematic review that has examined the endoscopic and EUS characteristics of colon SETs, where they evaluated 11 different types of colon SETs [6]. Therefore, this study aimed to identify diagnostic points for colon SETs by comparing histological findings and the endoscopic and EUS characteristics of incidentally diagnosed cases in over 100 cases.

## 2. Materials and Methods

### 2.1. Study Design, Setting, and Patients

This study was a retrospective cohort study of 194 patients who underwent EUS for suspicious colon SETs during colonoscopy at Samsung Medical Center from May 2014 to December 2021. A total of 135 lesions were assessed as colon SETs on EUS, and 105 were verified histologically (28 by simple forceps biopsies, 11 by bite-on-bite biopsies, and 66 by endoscopic mucosal resection). The other 30 were followed up without a biopsy. A total of 105 patients for whom tissue was obtained were finally enrolled, and their EUS findings were compared with the histological results (Figure S1). The study protocol was reviewed and approved by the Institutional Review Board at Samsung Medical Center (IRB No. 2022-10-101-001). The requirement to obtain informed consent was waived as we used only de-identified data routinely collected during hospital visits.

### 2.2. Instruments

An ultrasonic miniprobe (Olympus UM-2R, 12MHz; UM-3R, 20 MHz, Tokyo, Japan) was introduced under endoscope (Olympus CF-H260 or CF-Q260, Tokyo, Japan), as well as an endoscopic ultrasonography system (Olympus EU-ME2, Tokyo, Japan).

### 2.3. Procedures

The same technique used for traditional colonoscopy was used to prepare patients for EUS. When a raised lesion with normal mucosa was identified endoscopically, the tip of the colonoscope was positioned at the distal end of the lesion. The lumen was filled with 100–200 mL of water to achieve acoustic interaction between the transducer and the intestinal wall. The miniprobe was then introduced through the colonoscope’s working channel and progressed beyond the lesion. While the miniprobe was being moved across the lesion area, the lesion was evaluated by real-time ultrasonography. The normal colorectal wall is known to be visualized by EUS as a five-layered structure [16,17]. The identification of the SET layer of origin was achieved by examining the continuity between the lesion and the adjacent normal colonic wall. The location, surface color, surface change, consistency, size, echogenicity, internal echo, and the SET layer of origin were evaluated.

### 2.4. Variables and Definition

An incidental colon SET is a tumor identified beneath the epithelial surface during a medical checkup or screening for unrelated illnesses. These tumors are frequently detected incidentally, which means they were discovered by coincidence rather than as the primary focus of investigation.

We collected the following variables at the time of EUS: location, size, color, consistency, surface change, EUS layer of origin, echogenicity, and internal echo characteristics. Endoscopists can assess the consistency of an SET, whether soft or hard, by compressing the center with biopsy forceps. The brightness or reflectivity of the SET on the EUS was assessed as echogenicity (hypoechoic, hyperechoic, anechoic, isoechoic, or mixed), and homogeneity or uniformity of the SET on EUS was assessed as internal echo characteristics (homogenous or inhomogeneous).

Histopathology analysis was conducted by an experienced pathologic specialist in our medical center. The pathologic lesions were classified as malignant, potentially malignant, or benign lesions. We reviewed the endoscopic findings of each SET and compared the pathologic lesion analysis with detailed endoscopic and EUS findings.

The primary outcome was to set the diagnostic flow chart for evaluating colon SETs. We compared and analyzed the endoscopic and endoscopic ultrasound findings of each histologically diagnosed SET lesion and proposed an algorithm of diagnosing each SET lesion via differentiation points based on the endoscopic and endoscopic ultrasound findings.

### 2.5. Statistical Analysis

The values for categorical variables are expressed as the number and percentage. Categorical variables were analyzed using Fisher’s exact test. All statistical analyses were performed using SPSS Statistics, ver. 27.0 (IBM Corp, New York, NY, USA). A two-tailed *p* < 0.05 was considered to reflect a statistically significant result.

## 3. Results

### 3.1. Endoscopic and EUS Characteristics

The endoscopic and EUS characteristics of colon SETs were obtained in 105 patients and are summarized in Table 1. Among the colon SETs (*n* = 105), 32 were located in the transverse colon (30.5%), 20 in the sigmoid colon (19.0%), and 17 in the ascending colon (16.2%). The number of lesions less than 10 mm in size was 88 (83.3%). There were 63 (60.0%) pale lesions and 37 (35.2%) yellowish lesions. Ninety-six lesions had no surface changes (91.4%), and nine lesions (8.6%) had surface changes. Fifty-four lesions originated from the submucosal layer (51.4%) and sixteen from the muscularis mucosa layer (15.2%). Six cases (5.7%) originated from the both muscularis mucosa and submucosal layers, and twelve cases (11.4%) originated from both the submucosal and proper muscle layers. Among the echogenicity classifications, 56 were hypoechoic lesions (53.3%), 19 were hyperechoic lesions (18.1%), and 24 were mixed echoic lesions (22.9%). In the internal echo evaluation, 65 were homogeneous lesions (61.9%), and 40 were inhomogeneous lesions (38.1%).

### 3.2. Histopathologic Findings

The histologic diagnoses of colon SETs are shown in Table 2. Three cases (2.9%) were malignant, eight (7.7%) were potentially malignant, and most were benign (71/105, 67.6%) lesions. Two of the three malignant lesions were adenocarcinomas, and the other was lymphoma. Granular cell tumors (GCTs) (4.8%), neuroendocrine tumors (NETs) (1.9%), and perivascular epithelioid cell tumor (PEComa) (1.0%) were among the potentially malignant lesions. Lipomas, parasitic infections, lymphangiomas, and leiomyomas accounted for 18 (17.1%), 9 (8.6%), 7 (6.7%), and 6 (5.7%) of the benign lesions, respectively. A total of 23 out of 105 cases had pathological findings that were not diagnostic, such as focal lymphoid aggregates, non-specific colitis, or no significant pathologic alterations.

In particular, 17 out of the 28 (60.7%) cases with simple forceps biopsies showed no diagnostic value (Table S1). All five lesions subjected to endoscopic mucosal resection (EMR) were located in the SM layer. After EMR, it was established that the lesion had been eliminated using EUS, but only local lymphoid aggregates were observed on histological examination.

### 3.3. Endoscopic and Endosonographic Characteristics According to Histological Findings

**Figure 1:**
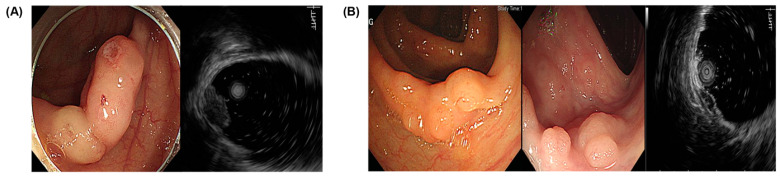
EUS findings of malignant lesions. (**A**) Adenocarcinoma and (**B**) lymphoma. EUS, endoscopic ultrasound.

**Figure 2:**
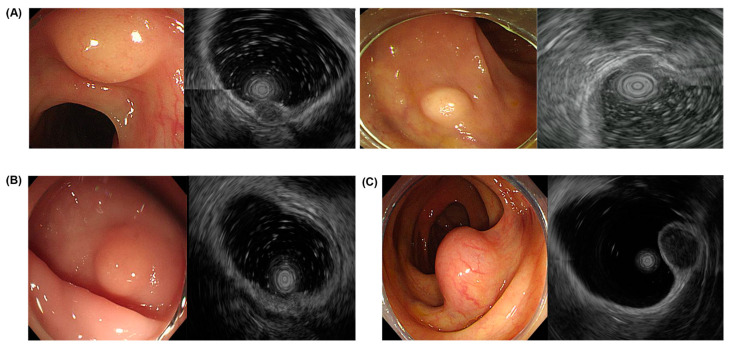
EUS findings of potentially malignant lesions. (**A**) Granular cell tumor, (**B**) neuroendocrine tumor, and (**C**) perivascular epithelioid cell tumor. EUS, endoscopic ultrasound.

Table 3 is a summary of the endoscopic and EUS findings connected to the histological findings. Adenocarcinomas had a hard consistency with ulcers on the surface and were revealed as hypoechoic, inhomogeneous masses located in the muscularis mucosa and submucosa in the EUS. Lymphoma had a hard consistency with nodular surface changes and was found as hypoechoic masses in the muscularis mucosa (Figure 1). GCTs and NETs had similar features, such as yellowish hypoechoic masses without surface changes. Colon NETs were found only in the rectosigmoid junction. PEComa measured 14.8 mm in size, had no surface alterations, and were found in the proper muscle layer with hypoechoic inhomogeneous features (Figure 2).

**Figure 3:**
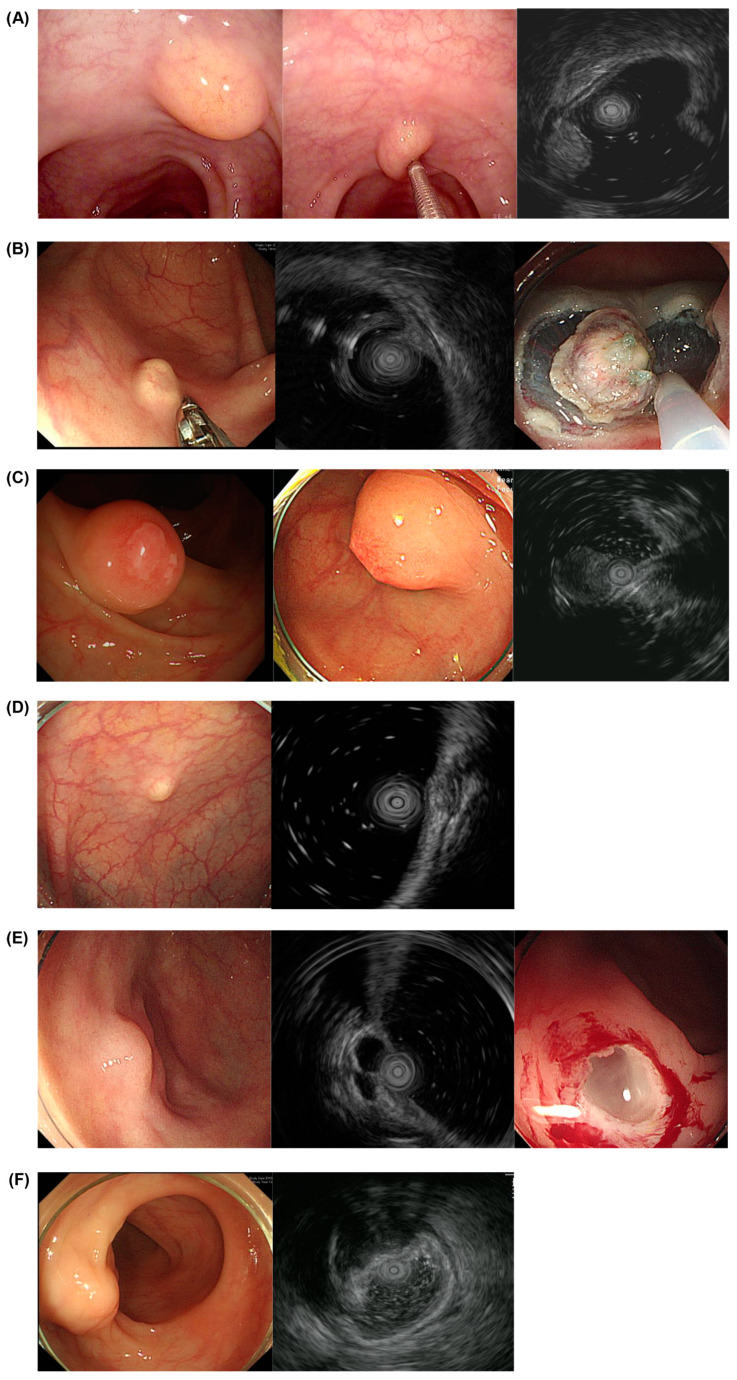
EUS findings of benign lesions. (**A**) Lipoma, (**B**) parasitic infection, (**C**) inflammatory fibroid polyp, (**D**) leiomyoma, (**E**) lymphangioma, and (**F**) endometriosis. EUS, endoscopic ultrasound.

Lipomas were yellowish and visualized as hyperechoic masses located in the submucosa. Most were soft, but 2 out of the 18 cases showed a hard consistency (one was accompanied by fat necrosis, and the other was accompanied by mucosal fibrosis). Parasitic infections and lesions resulting from various inflammatory reactions, such as fibrous/fibro-calcific lesions and necrotic nodules, were observed as mostly pale and hard SETs and revealed as mixed echogenic masses located in the muscularis mucosa, submucosa, or multi-layers in the EUS. When the parasitic infection lesion was accompanied by submucosal fat, it appeared yellowish endoscopically. Leiomyomas were displayed as hypoechoic masses from the mucosa to the muscularis propria. One leiomyoma lesion, which originated in the proper muscle layer, had a central hyperechoic nodule. Lymphangiomas were shown as submucosal anechoic or hypoechoic masses with a pale or transparent appearance. Endometriosis was pale and seen at multiple layers and was heterogeneous in echogenicity. One lesion was accompanied by nodular changes and surface redness (Figure 3).

Table 4 describes the endoscopic and EUS characteristics of malignant SETs as compared to non-malignant SETs. For malignant SETs, there was a statistically significant alteration in the surface of the tumor (*p* < 0.001), and they were located where the MM layer was included (*p* = 0.008). Although statistical significance was not established, all three malignant SET lesions located in the right colon were hard and pale and showed hypoechoic echogenicity in the EUS.

### 3.4. Diagnostic Approach for Incidental Colon SETs

**Figure 4:**
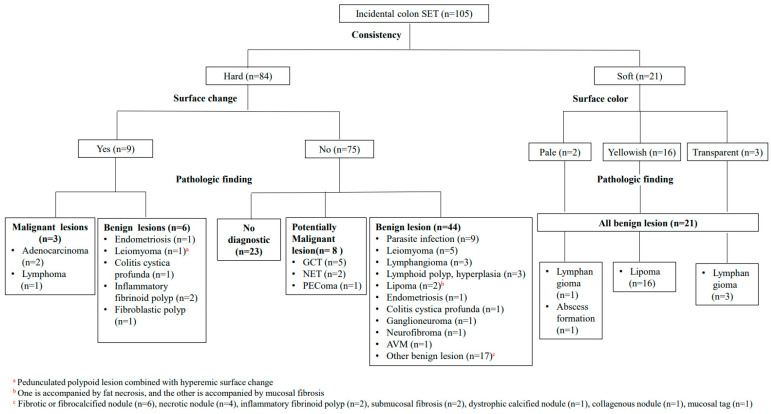
Diagnostic approach to incidentally found colon SETs. *SET*, subepithelial tumor; GCT, granular cell tumor; NET, neuroendocrine tumor; PEComa, perivascular epithelioid cell tumor; AVM, arteriovenous malformation.

The diagnostic classification approach for incidentally found colon SETs is described in Figure 4. First, out of the total 105 colonic SET lesions identified incidentally, 84 were categorized as hard, and 21 were classified as soft according to consistency. The 16 soft and yellowish lesions were lipomas; the 3 transparent lesions were lymphangiomas, and of the 2 pale lesions, 1 was a lymphangioma, and the other was an abscess formation. Three of the nine lesions with specific surface changes, such as ulcers or nodularity, were malignant, while the others were benign. If they were hard and had no surface changes, the majority of the lesions were benign (44/75, 58.6%) or had no diagnostic value (23/75, 30.7%), despite the fact that 76% (57/75) of the lesions were biopsied through bite-on-bite or EMR, and samples were taken deep enough. Only eight of the lesions (10.7%) were potentially malignant.

## 4. Discussion

Colon SETs are rare tumors, and no standardized diagnostic approach has been established. Notably, a characteristic endoscopic feature of colonic SETs is the presence of an elevated lesion with intact mucosa, each exhibiting distinct morphological characteristics. Colonoscopy is the most commonly used examination for the diagnosis of large intestine submucosal lesions. Several studies on the endoscopic morphologic features of colorectal submucosal tumors have also been conducted [15,18]. Nevertheless, limited published literature exists regarding formal guidelines and appropriate diagnostic approaches specifically tailored to colon SETs.

The location, color, surface feature, and hardness of the lesion can be identified through colonoscopy. A consistency check is a relatively simple method of pressing the lesion with forceps that can help differentiate the lesions. In this study, all soft lesions were confirmed to be benign lesions. Particularly, soft and yellow-colored lesions were determined to be lipomas, whereas soft and transparent lesions were revealed to be lymphangiomas. These findings provide confirmation that a hardness assessment can aid in distinguishing benign lesions to a certain extent. Specifically, when a lesion is determined to be soft, it indicates a higher probability of being benign.

In a review performed in 2005, the mean proportion of malignant lesions in a series of SETs was 12% (from 0 to 27%), and 8% were found in the colon and rectum [8]. The most important thing to do when an incidental SET lesion of the colon is found in clinical practice is to determine whether or not it is a malignant lesion. Malignancy is suspected when the size exceeds 3 cm, the margin is irregular, or the surface is accompanied by an ulcer, according to previous studies [6,8]. In our investigation, we observed that all three identified malignant lesions exhibited distinct characteristics, presenting as firm lesions with accompanying ulcers or nodular changes on the surface. Notably, our findings reveal a statistically significant difference in the surface characteristics between malignant and non-malignant SETs. Although there are rare malignant colon SETs, it is critical not to miss the malignant tumors. Therefore, a comprehensive examination of the surface of the lesion is required to avoid overlooking the malignant lesions when the SET is discovered by chance. Any observed alterations on the surface warrant additional investigations to accurately identify any malignant tumors.

It is difficult to determine the true size, origin layer, and histologic nature of a colon SET when using colonoscopy alone. The development of EUS has provided a completely new dimension to the diagnosis of colorectal lesions [19,20,21]. EUS plays a crucial role in visualizing and characterizing intramural tumors by providing information on their size, internal characteristics, contour, and primary layer of involvement. This enables a differential diagnosis, including the assessment of malignancy likelihood. The determination of the SET layer of origin is typically accomplished by employing a high-frequency miniprobe on the elevated area and establishing the continuity between the tumor and the colonic wall. Although endoscopy has limitations in distinguishing SETs from extramural compression (39–69%) [8], EUS was found to have a sensitivity of 92% and a specificity of 100% [22]. In this investigation, the colonoscopy findings suggest that 38 of 194 patients (19.6%) had an SET, whereas EUS tests revealed that they were extrinsic compression.

In this study, the majority of the SETs identified originated from the submucosal layers. Lipomas, lymphangiomas, and ganglioneuromas originated from the submucosal layers. In contrast, adenocarcinomas develop from the muscularis mucosa and submucosa, whereas lymphomas develop from the muscularis mucosa. Notably, while focusing on the malignant SETs, the muscularis mucosa was engaged in a significantly higher proportion than non-malignant SETs. It is critical to distinguish malignant SETs from non-malignant SETs, and the layer, in addition to the surface change, is a crucial component in this distinction. As a result, when there is involvement in the muscularis mucosa, careful examination of the risk for malignancies is required, emphasizing the importance of preventing overlooking rare malignant cases.

Echogenicity can also be helpful in the diagnosis of submucosal lesions. In this study, all lipomas, ganglioneuromas, and dystrophic calcified nodules were identified as hyperechoic masses. Mixed echoic masses encompassed parasitic infections, collagenous nodules, and lesions arising from various inflammatory processes, such as fibrous/fibro-calcific lesions and necrotic nodules. Lymphangiomas and colitis cystica profunda appeared as anechoic or hypoechoic masses. The majority of lesions, including adenocarcinomas, lymphomas, GCTs, NETs, and PEComa, exhibited a hypoechoic pattern. These observations suggest that hyperechoic, anechoic, or mixed echoic lesions are more indicative of benign lesions.

GGTs and NETs are recognized as potentially malignant lesions [14,23]. Both GGTs and NETs exhibited the characteristics of being yellowish and hard, with hypoechoic features observed in the muscularis mucosa and/or submucosal layer during the EUS examination. These similarities in appearance make it challenging to differentiate between the two without conducting a biopsy. However, NETs are known to be prevalent in the rectum [24], and two NETs identified in this study were revealed at the rectosigmoid junction. In contrast, GCTs were predominantly observed in the right colon [25], and in this study, GCTs were also discovered in the right-sided colon, such as the cecum, ascending colon, and transverse colon. These findings suggest that the location of the lesion can provide additional assistance in differentiation.

In the majority of cases, relying solely on EUS findings, especially in hypoechoic lesions, does not allow for a definitive diagnosis to be reached [1,8,26]. Histological diagnosis remains the most accurate diagnostic method for SETs. According to a prospective study comparing the diagnostic rates of forceps biopsies and the EMR of lesions in the submucosa, the forceps biopsy diagnostic rate was 17% (4 of 23), while the EMR diagnostic rate was 87% (20 of 23). The diagnostic rate based on EMR was much higher [5]. In fact, in this study, 23 of 105 total biopsies had no diagnostic value, such as nonspecific colitis, lymphoid aggregates, and no significant pathological alterations. The diagnostic yield varied among different biopsy techniques. The diagnostic rate was significantly lower for forceps biopsies, where 60.7% had no diagnostic value compared to 9.1% for bite-on-bite biopsies and 7.6% for EMR. These results suggest that forceps biopsies had a considerably lower diagnostic yield than the other biopsy techniques. All of the lesions that were not diagnostic were hard lesions with no surface change, and given the low diagnosis rate of forceps biopsy, at least a bite-on-bite biopsy was required in these lesions. On the other hand, given its high diagnosis rate, EMR is a fine option for diagnosing small SETs. Above all, EMR and endoscopic submucosal dissection (ESD) should be considered as the primary biopsy approach because a high diagnostic rate is essential when a malignant tumor is suspected.

Our study had certain strengths and limitations that should be considered. Being a retrospective study conducted in a single center, some degree of incomplete data collection was unavoidable. Furthermore, given that multiple gastroenterologists performed the EUS procedures in our medical center, there was inherent variability in image capture and lesion interpretation. However, we meticulously reviewed all the available data and images, aiming to minimize inter-observer variability through the expertise of experienced endoscopic specialists. Notably, this study represents the first comprehensive investigation of a large cohort comprising over 100 patients with colon SETs. Additionally, a significant strength lies in its unique contribution to the field by elucidating the differential diagnosis approach of colon SETs through a comparative analysis of histological, endoscopic, and EUS findings.

## 5. Conclusions

Based on our findings, the overwhelming majority of incidentally discovered small colon SETs tended to be benign. Despite their rarity, malignant SETs should not be overlooked. In cases where an SET exhibits surface ulceration or nodular changes, and it was placed where the MM layer was included on the EUS, pathological confirmation is critical given the risk of malignancy.

## Figures and Tables

**Table 1:** Endoscopic and EUS characteristics of colon SETs (*N* = 105).

Characteristics	No. (%)
**Location**	
Cecum	16 (15.2%)
Ascending colon	17 (16.2%)
Hepatic flexure	6 (5.7%)
Transverse colon	32 (30.5%)
Splenic flexure	2 (1.9%)
Descending colon	3 (2.9%)
Sigmoid descending junction	2 (1.9%)
Sigmoid colon	20 (19.0%)
Rectosigmoid junction	7 (6.7%)
**Size (mm)**	
<10	88 (83.8%)
≥10	17 (16.2%)
**Consistency**	
Hard	84 (80%)
Soft	21 (20%)
**Color**	
Pale	63 (60.0%)
Yellowish	37 (35.2%)
Transparent	5 (4.8%)
**Surface change**	
No	96 (91.4%)
Yes	9 (8.6%)
**EUS layer of origin**	
Muscularis mucosa (MM)	16 (15.2%)
Submucosa (SM)	54 (51.4%)
Proper muscle (PM)	17 (16.2%)
MM + SM	6 (5.7%)
SM + PM	12 (11.4%)
**Echogenecity**	
Hypoechoic	56 (53.3%)
Hyperechoic	19 (18.1%)
Anechoic	4 (3.8%)
Isoechoic	2 (1.9%)
Mixed	24 (22.9%)
**Internal echo**	
Homogeneous	65 (61.9%)
Inhomogeneous	40 (38.1%)

EUS: endoscopic ultrasound; SETs: subepithelial tumors; MM: muscularis mucosa; SM: submucosa; PM: proper muscle.

**Table 2:** Histopathology of colon SETs (*N* = 105).

Pathology	No. (%)
**Malignant lesion**	
Adenocarcinoma, well differentiated	2 (1.9%)
Lymphoma	1 (1.0%)
**Potentially malignant lesion**	
Granular cell tumor	5 (4.8%)
Neuroendocrine tumor	2 (1.9%)
Perivascular epithelioid cell tumor	1 (1.0%)
**Benign lesion**	
Lipoma	18 (17.1%)
Parasitic infection	9 (8.6%)
Lymphangioma	7 (6.7%)
Leiomyoma	6 (5.7%)
Fibrotic or fibrocalcified nodule	5 (4.8%)
Inflammatory fibrinoid polyp	4 (3.8%)
Necrotic nodule	4 (3.8%)
Lymphoid polyp or hyperplasia	3 (2.9%)
Endometriosis	2 (1.9%)
Colitis cystica profunda	2 (1.9%)
Submucosal fibrosis	2 (1.9%)
Ganglioneuroma	1 (1.0%)
Neurofibroma	1 (1.0%)
Fibroblastic polyp	1 (1.0%)
Fibrous tumor	1 (1.0%)
Dystrophic calcified nodule	1 (1.0%)
Collagenous nodule	1 (1.0%)
Arteriovenous malformation (AVM)	1 (1.0%)
Abscess formation	1 (1.0%)
Mucosal tag	1 (1.0%)
**No diagnostic value**	23 (21.9%)
**Total**	105 (100%)

SETs: subepithelial tumors.

**Table 3:** Endoscopic and EUS findings of colon SETs connected to the histological findings.

Lesions		Endoscopic Finding			EUS Finding	
		Consistency	Surface Change	Surface Color	Layer	Echogenecity
Malignant	Adenocarcinoma	Hard	Ulcer	Pale	Muscularis mucosa and submucosa	Hypoechoic (inhomogenous)
	Lymphoma	Hard	Nodular changes	Pale	Muscularis mucosa	Hypoechoic
Potentially malignant	Granular cell tumor	Hard	No	Yellowish	Muscularis mucosa or submucosa or multilayer	Hypoechoic
	Neuroendocrine tumor	Hard	No	Yellowish	Muscularis mucosa or submucosa	Hypoechoic
	Perivascular epithelioid cell tumor	Hard	No	Pale	Proper muscle	Hypoechoic (inhomogenous)
Benign	Lipoma	Mostly soft ^a^	No	Yellowish	Submucosa	Hyperechoic
	Parasitic infection	Hard	No	Mostly pale ^b^	Muscularis mucosa or submucosa or multilayer	Mixed echogenicity
	Leiomyoma	Hard	No	Pale	Muscularis mucosa or submucosa or proper muscle	Hypoechoic ^c^
	Endometriosis	Hard	No or nodular change-combined hyperemia	Pale	Multilayer	Mixed echogenicity
	Lymphangioma	Hard or soft	No	Pale or transparent	Submucosa	Anechoic or hypoehoic
	Colitis cystica profunda	Hard	No	Pale or transparent	Muscularis mucosa or submucosa	Anechoic or hypoehoic (inhomogenous)
	Ganglioneuroma	Hard	No	Pale	Submucosa	Hyperechoic
	Neurofibroma	Hard	No	Pale	Muscularis mucosa	Hypoehoic (inhomogenous)
	Arteriovenous malformation	Hard	No	Pale	Submucosa	Hypoehoic (inhomogenous)
	Lymphoid polyp or hyperplasia	Hard	No	Pale	Muscularis mucosa	Hypoehoic
	Fibrotic or fibrocalcified nodule	Hard	No	Pale or yellowish	Muscularis mucosa or submucosa or proper muscle	Mixed or hypoehoic (inhomogenous)
	Necrotic nodule	Hard	No	Pale or yellowish	Submucosa or proper muscle	Mixed echogenicity
	Inflammatory fibrinoid polyp	Hard	No or hyperemic change	Pale	Submucosa or proper muscle or multilayer	Mixed or hypoehoic (inhomogenous)
	Dystrophic calcified nodule	Hard	No	Yellowish	Submucosa	Hyperechoic
	Collagenous nodule	Hard	No	Pale	Submucosa and proper muscle	Mixed echogenicity

^a^ Two cases showed hard consistency (one is accompanied by fat necrosis, and the other is accompanied by mucosal fibrosis). ^b^ When accompanied by submucosal fat, it may appear yellowish endoscopically. ^c^ Leiomyoma originated in the PM layer has a central hyperechoic nodule. EUS: endoscopic ultrasound; SETs: subepithelial tumors; MM: muscularis mucosa; SM: submucosa; PM: proper muscle.

**Table 4:** Endoscopic and EUS characteristics associated with malignant SETs.

Characteristics	Malignancy (*n* = 3)	Non-Malignancy (*n* = 102)	*p*-Value
**Location**			0.55
Right colon	3 (100%)	68 (66.7%)	
Left colon	0 (0%)	34 (33.3%)	
**Size (mm)**			0.42
<10	2 (66.7%)	86 (84.3%)	
≥10	1 (33.3%)	16 (15.7%)	
**Consistency**			1.00
Hard	3 (100%)	81 (79.4%)	
Soft	0 (0%)	21 (20.6%)	
**Color**			0.39
Pale	3 (100%)	60 (58.8%)	
Yellowish	0 (0%)	37 (36.3%)	
Transparent	0 (0%)	5 (4.9%)	
**Surface change**			<0.001
No	0 (0%)	96 (94.1%)	
Yes	3 (100%)	6 (5.9%)	
**EUS layer of origin**			0.008
Including muscularis mucosa (MM) ^a^	3 (100%)	19 (18.6%)	
Others ^b^	0 (0%)	83 (81.4%)	
**Echogenecity**			0.65
Hypoechoic	3 (100%)	53 (52.0%)	
Hyperechoic	0 (0%)	20 (19.6%)	
Anechoic	0 (0%)	4 (3.9%)	
Isoechoic	0 (0%)	2 (2.0%)	
Mixed	0 (0%)	23 (22.5%)	
**Internal echo**			0.56
Homogeneous	1 (33.3%)	64 (62.7%)	
Inhomogeneous	2 (66.7%)	38 (37.2%)	

^a^ MM (*n* = 16) MM+SM (*n* = 6). ^b^ SM (*n* = 54), PM (*n* = 17), and SM + PM (*n* = 12). EUS: endoscopic ultrasound; SETs: subepithelial tumors; MM: muscularis mucosa; SM: submucosa; PM: proper muscle.

## Data Availability

The data underlying this article cannot be shared publicly, given the privacy of the individuals who participated in the study. The data will be shared upon reasonable request to the corresponding author.

## Supplementary Materials

The following supporting information can be downloaded at https://www.mdpi.com/article/10.3390/diagnostics14050551/s1, Figure S1: Patient flow chart.; Table S1: Diagnosis rate according to each biopsy method.
