# Supplementary material for: Clinicopathologic and Endosonographic Characteristics of Colon Subepithelial Tumors Discovered Incidentally
Source: Diagnostics (Basel). 2024 Mar 5;14(5):551. doi: 10.3390/diagnostics14050551 (PMC10930823; doi:10.3390/diagnostics14050551)
Supplement: Supplementary file 1 [file diagnostics-14-00551-s001.zip › diagnostics-2837906-supplementary.pdf]

Table S1. Diagnosis rate according to each biopsy method.

	No diagnostic	Diagnostic
Forcep biopsy	17/28 (60.7%)	11/28 (39.3%)
Bite on bite biopsy	1/11 (9.1%)	10/11 (90.9%)
Endoscopic mucosal resection (EMR)	5/66 (7.6%)	61/66 (92.4%)
Total	23/105 (21.9%)	82/105 (78.1%)

Figure S1. Patient flow chart.

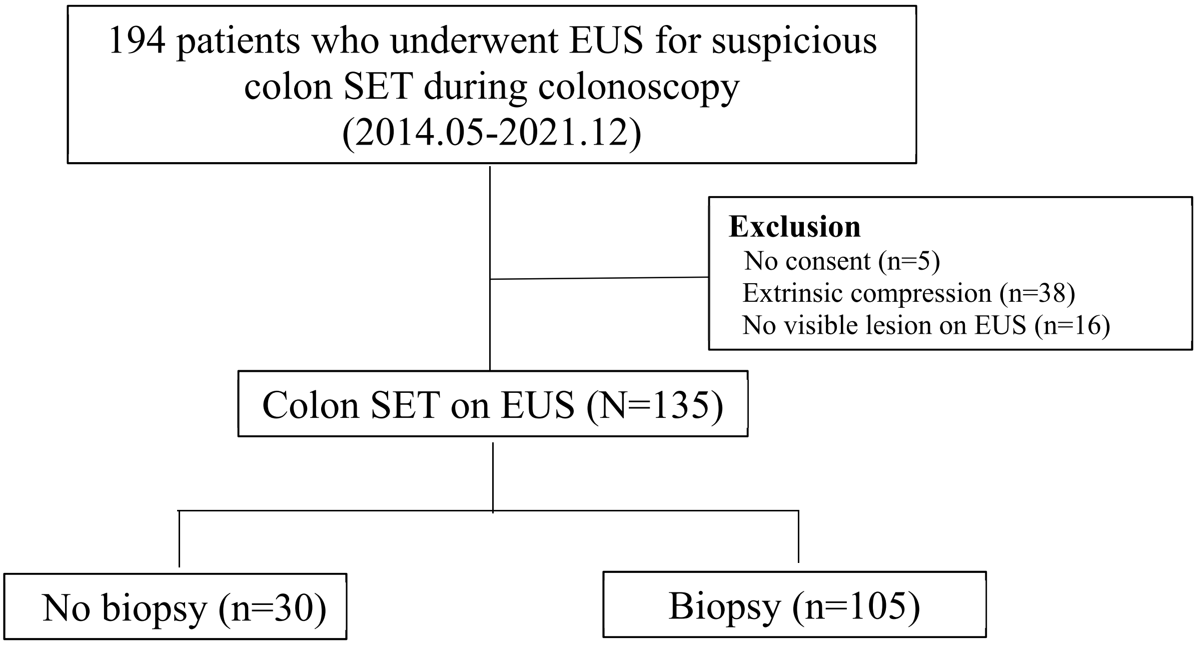

EUS: endoscopic ultrasound; SET: subepithelial tumor.
